# Phytochemical Composition and Antibacterial Activity of Hydroalcoholic Extracts of* Pterospartum tridentatum* and* Mentha pulegium* against* Staphylococcus aureus* Isolates

**DOI:** 10.1155/2016/5201879

**Published:** 2016-04-14

**Authors:** Alfredo Aires, Eduardo Marrinhas, Rosa Carvalho, Carla Dias, Maria José Saavedra

**Affiliations:** ^1^Centre for the Research and Technology for Agro-Environment and Biological Sciences, CITAB, University of Trás-os-Montes e Alto Douro, Quinta de Prados, 5001-801 Vila Real, Portugal; ^2^University of Trás-os-Montes e Alto Douro, UTAD, Quinta de Prados, 5001-801 Vila Real, Portugal; ^3^Animal and Veterinary Research Centre, CECAV, University of Trás-os-Montes e Alto Douro, Quinta de Prados, 5001-801 Vila Real, Portugal

## Abstract

*Pterospartum tridentatum* and* Mentha pulegium* are largely used in Portuguese folk medicine to treat several human disorders and inflammatory processes but without any consistent evidence for those beneficial pointed properties. Thus, the aim of the current work is to evaluate its benefits and phytochemicals related to those beneficial properties. A distinct polyphenol profile between* P. tridentatum* and* M. pulegium* was found. Taxifolin, myricetin, ginestin, ginestein, and ginestein derivatives, biochanin A-glucoside, and biochanin A were identified in* P. tridentatum*, whilst in* M. pulegium* the luteolin-7-rutinoside, diosmin, and apigenin and respective derivatives were most representative polyphenols. These variations had implications in the antiradical and antibacterial activity and the* P. tridentatum* exhibited the highest antibacterial activity against methicillin-resistant and methicillin-sensitive* Staphylococcus aureus* MSSA, which was mainly dose-dependent. This antibacterial activity seems to be related to high content of flavonols, flavones, and isoflavones, which can act synergistically with each other against this type of bacteria. Our results showed consistent evidence that* Pterospartum tridentatum* and* Mentha pulegium* are an important reservoir of phytochemicals with antiradical activity and antibacterial capacity and thus they might be used in a preventive way or in a combined pharmaceutical and antibiotic therapy against pathogenic bacteria.

## 1. Introduction

Nowadays, one key problem in human health is the less effectiveness of commercial antibiotics against several pathogenic bacterial isolates. One of them is the* Staphylococcus aureus*, a gram-positive bacterium from Staphylococcaceae family, and considered one of the world's most important infectious agents causing disease outbreaks related to food consumption, badly treated wounds, and hospital-associated infections [1, 2].* S. aureus* is often reported as being for a variety of human and animal diseases and its epidemiological relevance is mainly due to their ability of becoming highly resistant to common antimicrobials such as tetracycline, vancomycin, penicillin G, and methicillin [3, 4] and to a less degree to oxacillin, lincomycin, clindamycin, erythromycin, streptomycin, cefoxitin, kanamycin, chloramphenicol, and gentamicin [5, 6].

In the last decades, evolution of resistance, for example, to methicillin, has become an enormous problem for treatment of* S. aureus* infections. Thus, the health authorities have increased the research programs to develop new and more effective antimicrobial molecules and several plants have been used in different ways to extract potential antimicrobial compounds. Different authors have shown that plants have naturally bioactive compounds that could act alone or in synergy with antibiotics against bacterial isolates [7, 8] and the aromatic and medicinal plants have been one of the most studied plants and found useful as antibacterial, antifungal, and antihelminthic [9–11] among other beneficial properties. However there is still a lack of information about either their phytochemical composition, their antimicrobial activity, or even how the phytochemicals act against microorganisms. Two common plants highly present in native flora of Mediterranean areas particularly in the Iberian Peninsula are the* Mentha pulegium*, normally called European pennyroyal, and* Pterospartum tridentatum* (L.) W. K. & Lge. (Syn.:* Genista tridentata* L. subsp.* cantabrica* (Spach) Nyman), frequently named as “*Carqueja*.”* M. pulegium* is an aromatic herb that belongs to the family Lamiaceae, naturalized in America, and thrives in western, southern, and central Europe, Asia, Iran, Arab countries, and Ethiopia [12]. Its essential oil and dry parts have been traditionally used in medicine (digestive, liver, and gallbladder disorders, amenorrhea, gout, colds, increased micturition, skin diseases, and abortifacient), gastronomy (culinary herb), aromatherapy, and cosmetics [13].* P. tridentatum* is a small shrub belonging to the Leguminosae family and Papilionoideae subfamily [14], and its flowers are traditionally used in folk medicine as depurative and hypoglycaemic and for throat irritation conditions [15]. Most studies performed so far on* M. pulegium* and related* Mentha* species were carried out with their essential oil in different regions of the world, including Tunisia [16], Greece [17], Turkey [18], and Portugal [19], and focused mainly on their chemical composition. There is still a lack of information about their phytochemical composition related to functional capacity and antimicrobial activity. Thus, we set this study in which we evaluate the phytochemical composition of* P. tridentatum* and* M. pulegium* and its effect on the antioxidant activity and antimicrobial potential against different isolates of* Staphylococcus aureus*, an important pathogen highly associated with outbreak diseases and antibiotic resistance phenomena.

## 2. Material and Methods

### 2.1. Sampling

The sampling process was done according to previous works conducted in our lab and already published [20], but with the same modifications. One kilogram of fresh* Pterospartum tridentatum* and* Mentha pulegium* was collected in natural open fields in Portugal. The* P. tridentatum* (*Carqueja*) samples were collected in Vila Real Region (altitude of 400 meters) near the Natural Park of Alvão (Northern Portugal) (N 41°17′35.538′′, W 7°44′29.6268′′), whilst* M. pulegium* (pennyroyal) samples were collected in Santarem Region (Central Portugal). These species are largely present in open fields of Portugal as native flora, but in the northern region* Carqueja* is more common whilst in the south the predominance goes to* Pennyroyal*. After harvest, the samples were botanically identified by the Botany Services of University of Trás-os-Montes and Alto Douro (UTAD), Vila Real, Portugal. After this identification, fresh samples were dried in a freeze-dryer system (UltraDry Systems*™*, USA), milled and reduced to a fine powder, and stored in dark flasks at 4°C in a dark environment until extraction. Fresh and dry weights were registered and the level of dry matter was determined.

### 2.2. Extraction

Dried powder of each sample (200 g) was extracted in triplicate with methanol 70% (methanol : water, v/v) in a warm bath at 70°C in 30 minutes with intermittent agitation. After that, methanolic extracts were filtered (Whatman No. 1), centrifuged at 4000 rpm during 15 min 4°C (Kubota, 2000). Hydroalcoholic extracts were then evaporated until complete dryness in a rotary evaporator under vacuum (40°C, 178 mbar). Yields of extraction were calculated. The final concentration achieved was 5 mg·mL^−1^ dry weight. The concentration was prepared diluting the solid residue with dimethyl sulfoxide (DMSO) (Sigma-Aldrich, Taufkirchen, Germany). These extracts were used for the phytochemical analysis and in vitro bioassays of antioxidant and antimicrobial activity.

### 2.3. Phytochemical Analysis HPLC-DAD-UV/VIS

Polyphenols generally named phenolic compounds are the group of secondary metabolites frequently found in extracts of aromatic and medicinal plants and they are often reported as having antimicrobial and antioxidant activity. The identification and quantification of phenolics in* P. tridentatum* and* M. pulegium* samples were performed using HPLC-DAD-UV/VIS. From the previous extracts, an aliquot of 1 mL was overnight evaporated until complete dryness under continuous nitrogen flux. The dried extracts were then resuspended with 1 mL of 70% methanol (methanol : water, v/v) and filtered (Spartan Ø 0.13) to HPLC amber vials (to avoid degradation by light) and stored at −20°C until injection in HPLC. The eluent was composed of water with 1% of trifluoroacetic acid (TFA) (solvent A) and acetonitrile with 1% TFA (solvent B). Elution was performed at a flow rate of solvent of 1 mL·min^−1^, with gradient starting with 100% of water, and the injection volume of 20 *μ*L. Chromatograms were recorded at 280, 320, 370, and 520 nm with a C18 column (250 × 46 mm, 5 *μ*m). Phytochemicals were identified using peak retention time, UV spectra, and UV max absorbance bands and through comparison with external commercial standards (Extrasynthese, France). The external standards were freshly prepared in 70% methanol (methanol : water) in a concentration of 1.0 mg·mL^−1^ and analysed by HPLC-DAD-UV immediately before the samples.. Methanol and acetonitrile were purchased from Panreac Chemistry (Lisbon, Portugal) and Sigma-Aldrich (Taufkirchen, Germany), respectively. The aqueous solutions were prepared using ultrapure water (Milli-Q, Millipore).

### 2.4. Evaluation of Functional Properties

#### 2.4.1. Free Radical Scavenging of 2,1-Diphenyl-2-picrylhydrazyl Free Radical (DPPH^•^)

The scavenging capacity of DPPH of methanolic* P. tridentatum* and* M. pulegium* extracts was determined using a spectrophotometric 96-well microplate assay [21] with several modifications as follows: 20 *μ*L of sample extract and 280 *μ*L of 60 *μ*M methanolic radical DPPH solution freshly prepared added to each well. Then the plate was left to stand at room temperature in 30 min. After that, the reduction in absorbance was measured at 517 nm with a spectrophotometer (Multiskan*™* FC Microplate Photometer, USA). The free radical scavenging activity as expression of antioxidant activity (AA) was calculated as percentage inhibition of the DPPH radical, according to the following equation: *I* (%) = [((solvent absorbance − sample absorbance)/solvent absorbance) × 100]. The compound butylated hydroxytoluene (BHT) (Sigma-Aldrich, Taufkirchen, Germany), a synthetic analog of vitamin E, was used as positive control of AA in order to compare the results for the samples. Also the inhibition concentration at 50% inhibition (IC_50_) was determined in order to compare the AA between the extracts themselves and extracts and positive control, and lower IC_50_ means better free radical scavenging activity, thus higher AA. All determinations were performed in triplicate.

#### 2.4.2. In Vitro Antibacterial Activity

*(1) Bacterial Isolates*. Seven gram-positive isolates of* Staphylococcus aureus* (3 methicillin-resistant* Staphylococcus aureus* and 3 methicillin-sensitive* Staphylococcus aureus* MSSA, plus one standard control strain from the American type culture collection (ATTC)) were obtained from Maria José Saavedra (Ph.D.) core collection located in Microbiology Laboratory of Veterinarian Science Department of UTAD (Table 1). The isolates were previously and properly identified by standard biochemical classification techniques [22] using API 20E, API 20NE, API Staph-Ident, and API Step (BioMerieux), according to the procedure previously described [23], followed by genetic identification through 16S rRNA sequencing. When tested, the isolates were prepared freshly, sowed in Petri plates (92 × 16 mm, Sarstedt, Germany) with BHI (Brain Heart Infusion, Oxoid, England) media, and incubated at 37°C overnight in order to obtain fresh and pure bacteria cultures. Then, bacterial suspension (5 × 10^6^ cfu·mL^−1^) of each isolate was prepared adjusting to an optical density range of 0.5–1.0 measured at OD_620 nm_ and used in the bioassays in order to obtain in each well 5 × 10^5^ cfu·mL^−1^ inoculum final concentration.

*(2) Determination of a Minimum Inhibitory Concentration (MIC)*. Phytochemicals are routinely classified as antimicrobials on the basis of susceptibility assays that produce a MIC within the range of 100–1000 *μ*g·mL^−1^ [24] and the MIC was defined as the lowest concentration of an antimicrobial compound which can maintain or reduce the growth of a microorganism after 24 hours of incubation [25]. In the current study, the antibacterial activity was assessed by MIC and the* P. tridentatum* and* M. pulegium* methanolic extracts were prepared with a maximum concentration of 5000 *μ*g·mL^−1^ dry weight in 10% DMSO in a microplate bioassay [26]. After that, 100 *μ*L of each extract and 1000 *μ*g of standard antibiotic were added into the first row of 96-well microplates, followed by serial dilutions on the additional wells containing 100 *μ*L of nutrient broth. Positive and negative controls were included: a column with gentamicin (Oxoid, England) as positive control and three negative controls (a column without bioactive compounds, a column without the bacterial solution (20 *μ*L of nutrient broth instead), and a column with DMSO solution). After this mixture the optical densities (OD) were measured at 620 nm (Multiskan FC Microplate Photometer, USA) that were automatically recorded. Then, the microplates were placed at 37°C during 24 hours and then the OD were measured again at 620 nm. These absorbance values were subtracted from those obtained before incubation to deplete the effect of color interference. The MIC was considered the lowest concentration in which the final OD was inferior to the initial OD. To classify the antibacterial effect we adopted the following scale: strong (+++), if MIC values ≤100 *μ*g·mL^−1^, moderate (++) when 100 < MIC ≤ 500 *μ*g·mL^−1^, weak (+) when 500 < MIC ≤ 1000 *μ*g·mL^−1^, and null (−) (without effect) when MIC > 1000 *μ*g·mL^−1^.

### 2.5. Statistical Analysis

The results were expressed as mean values and standard deviation (SD) of three replicates. The results were analyzed using one-way ANOVA followed by Duncan multiple range test, based on confidence level equal to or higher than 95% (*p* < 0.05). Software SPSS V.17 (SPSS-IBM, Orchard Road, Armonk, New York, NY, USA) was used to carry out this analysis.

## 3. Results and Discussion

**Figure 1:**
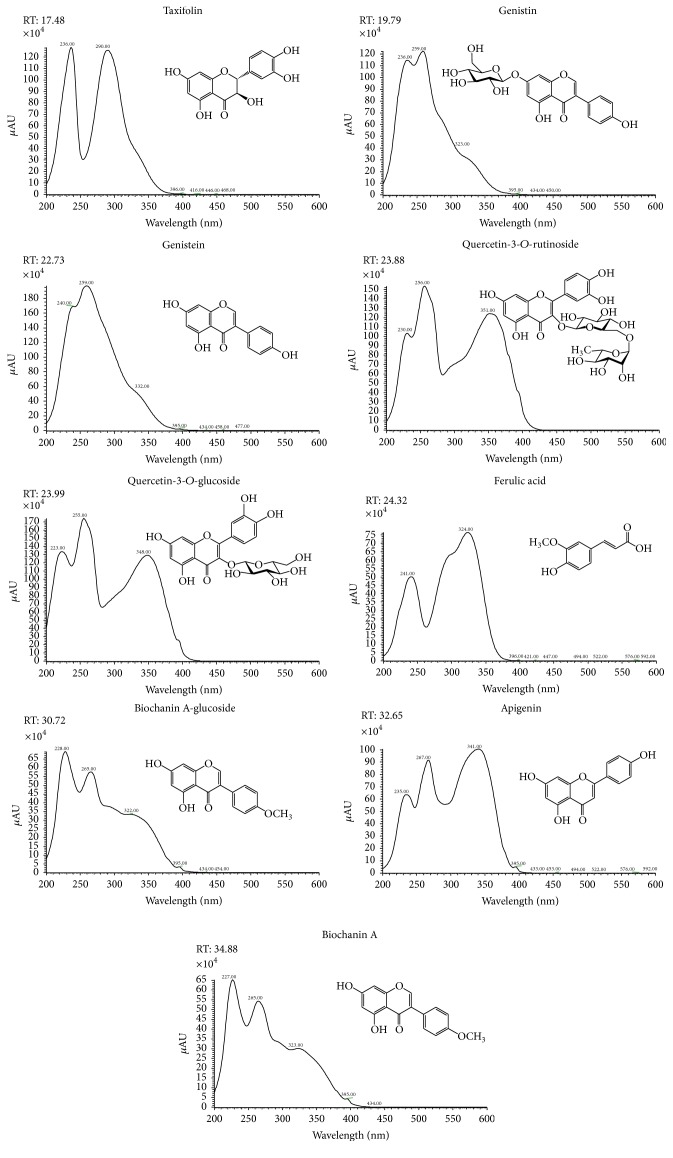
UV spectra of polyphenols detected in* Pterospartum tridentatum*.

**Figure 2:**
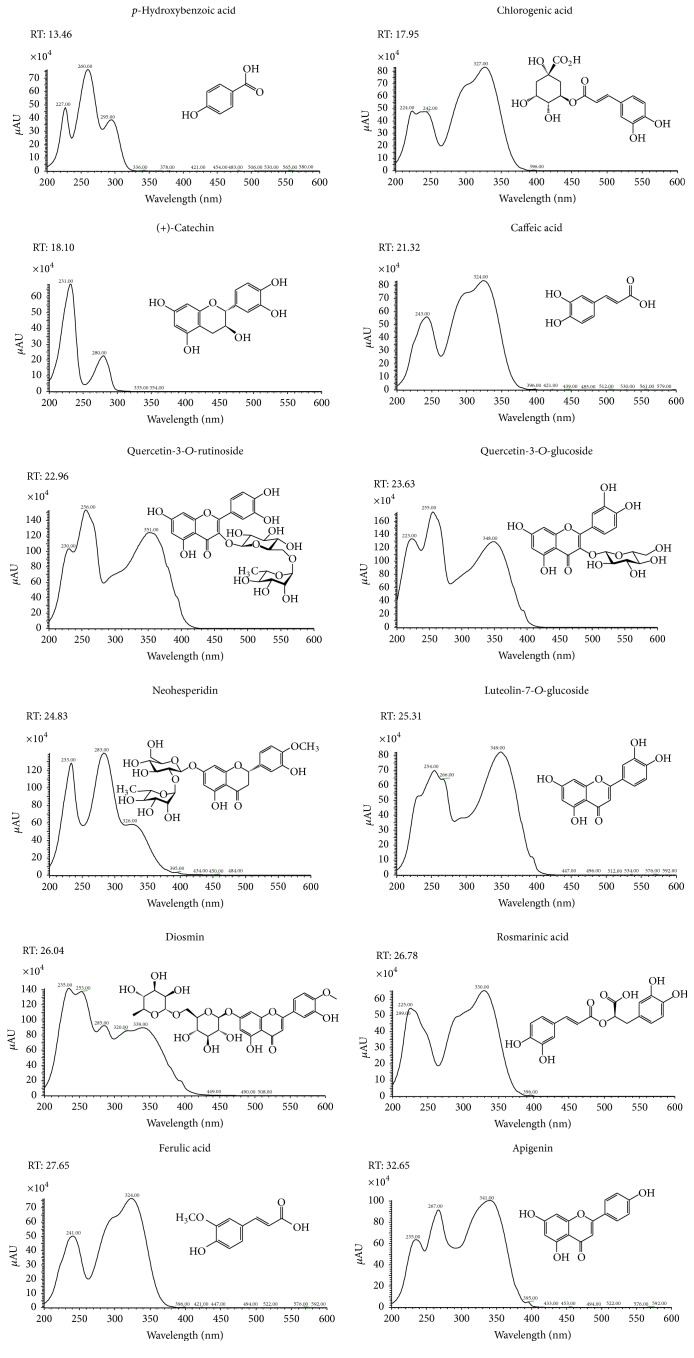
UV spectra of polyphenols detected in* Mentha pulegium*.

The polyphenol profile and respective chemical structures of phenolics identified in the current study of hydroalcoholic extracts from* P. tridentatum* and* M. pulegium* are presented in Figures 1 and 2. The respective data (retention time, *λ*_max_ in the visible region) and average content of each polyphenol, expressed as mg·g^−1^ dry weight (dw), are presented in Tables 2 and 3. It was possible to assess that* P. tridentatum* had high content taxifolin, ginestin, ginestein, and ginestein derivatives, biochanin A-glucoside, and biochanin A, whilst* M. pulegium* exhibited higher diversity with luteolin-7-rutinoside, diosmin, and apigenin and respective derivatives have the most representative phenolics identified.* P. tridentatum* was richer in isoflavones, whilst* M. pulegium* present high but similar content in flavones and hydroxycinnamic acids. These results have shown an important class of phytochemicals in both plant species. The presence of high levels of taxifolin, ginestin, and ginestein, all frequently associated with antioxidant, antimicrobial, and anthelmintic activity [27–31], made this plant extract very interesting from bioactive point of view. Taxifolin, a flavanol subclass of flavonoids [28], is abundant in several types of plants and is an interesting potential component of dietary supplements or antioxidant-rich functional food [29]. Genistein is an isoflavone [28] known by its anti-inflammatory and antioxidant properties [30] and has been shown to interact with animal and human estrogenic receptors. This compound is often mentioned as responsible for wound healing properties [31], which is one of the main reasons because plants with high content of such compounds are largely used in traditional medicine, even if in the majority of the cases there is no scientific evidence for that. In fact, native floras have been used in folk medicine for thousands years, even if their biological effects and chemicals responsible for those properties were poorly understood. Only in the recent years, the therapeutic value of several herbs and the direct relation between their bioactive potential and their content in some specific essential oils (EOs), alkaloids, terpenoids, glucosinolates, and phenolics, among other compounds, were properly established. Our results showed that* P. tridentatum* and* M. pulegium* have important phenolics often associated with anti-inflammatory, antioxidant, and antimicrobial properties [32], reinforcing the scarce information available until now about these two herbs. Works about the phytochemical composition of these two native plants are very scarce but some of them [32, 33] seem to be in agreement with our results. Quercetin, genistein, and bioachanin-A and related isomers have been found by other authors in* P. tridentatum* [32], whilst in different types of* Mentha* [33] the presence of catechin and catechins derivatives, rosmarinic acid, quercetin, luteolin, and apigenin was detected as we found in this study. This difference could be related not only to genetic factors but also to agroclimatic conditions, since these herbs were collected in two different Portuguese regions: the* P. tridentatum* was collected in Northern Portugal (more temperate and wet) whilst* M. pulegium* was collected in Centre-South of Portugal (more dry and hot). As consequence, both profile and average content of phenolics of these two herbs were different. This difference may explain the difference noted in their bioactivity.

**Figure 3:**
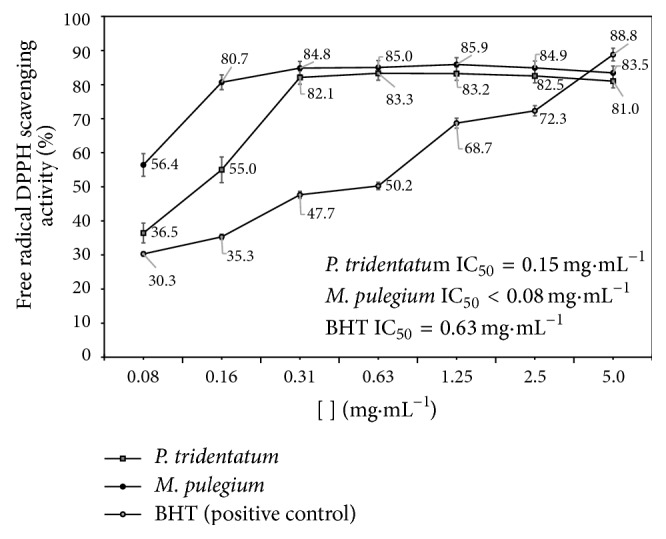
% free radical DPPH scavenging activity of* Pterospartum tridentatum* and* Mentha pulegium* methanolic extracts.

The results for the evaluation of functional properties expressed as free radical (2,1-diphenyl-2-picrylhydrazyl free radical (DPPH^•^)) activity and in vitro antibacterial activity are presented in Figure 3 and in Table 4, respectively. We observed that biological* P. tridentatum* and* M. pulegium* have high levels of AA, even higher than the positive control (BHT) used in this assay (Figure 3). Despite this similarity, a different trend in the phenolic influence on AA was observed. In the* P. tridentatum* the high AA seems to be explained by the high content of isoflavones and flavanols (49 and 42% of total phenolics identified, resp.), whilst in* M. pulegium* the AA seems to be explained by the synergism between phenolics acids (32% of total phenolics), flavones, and flavanones (31 and 17% of total phenolics, resp.). This difference is understandable since all of these compounds are often reported [27, 29, 30, 32, 33] as having important antioxidant activities. The presence of several phenolic acids, flavones, isoflavones, and flavanols in both extracts, with potential to scavenge free radicals such as superoxide and nitric oxide [29], can thus explain the high antioxidant potential exhibited by these two herbs. However, based on the average content of phenolics (Tables 2 and 3) the expectation was to have higher AA in* P. tridentatum* extracts but was not the case. In fact, based on the values of IC_50_ (Figure 3) the AA in* M. pulegium* was higher compared to* P. tridentatum*, which seems to contradict the previous finding, but we must be aware that other compounds, besides the phenolics such as pigments, alkaloids, and carotenoids (not determined in this work), often reported as being present in high concentrations in these two herbs [34], might have contributed to high levels of AA observed in this type of extract. In addition, the higher presence of phenolic acids such as hydroxybenzoic acid, chlorogenic acid, caffeic acid, ferulic acid, and rosmarinic acid, all based on –CH=CH–COOH groups, widely recognizable for forming easily complexes with DPPH [35] seems to be one of the main reasons for* M. pulegium* extracts having the lowest IC_50_ and thus the highest AA. Therefore, based on the current work it seems that the higher proportion of phenolic compounds with hydroxyl groups on the aromatic ring is responsible for the higher AA exhibited by* M. pulegium* extracts, which is in agreement with the previous findings [35] in which the positions of hydroxyl groups were found extremely important for the bioactivity of polyphenols, including their antioxidant capacity. Despite these differences, the high AA found for both extracts are critical and determinant for their therapeutic value and this may be in part responsible for their reputation as anti-inflammatory, hypotensive, hypoglycemic, and depurative agent. It seems that they can reduce oxidative stress, a key factor in the progression of chronical inflammatory diseases. Further investigations should be done in order to determine the action keys on the pathways of the inflammatory mechanisms. Based on these results it seems very clear that the traditional usage of* P. tridentatum* and* M. pulegium* as medicinal plants associated with anti-inflammatory and depurative processes is correct and thereby these plants serve as natural sources of antioxidants for food and medicinal purposes.

Although the results with DPPH^•^ free radical scavenging activity have shown that* P. tridentatum* and* M. pulegium* have similar values of AA, the antibacterial activity was very different.* P. tridentatum* exhibited the highest antibacterial activity due to lower minimum inhibitory concentration levels found. The MSSA isolates were more affected than MRSA isolates, as we expected. This activity was mainly dose-dependent and in general according to the classification criteria adopted (Table 4) the antibacterial activity was strong and in some isolates similar to the antibacterial activity observed for the antibiotic used as positive control. As similar to AA, the higher antimicrobial activity for* P. tridentatum* can be the consequence of two effects: (i) the higher content of flavonols and isoflavones and (ii) the additive effect of different types of phenolics, which seems to boost the antimicrobial efficacy of such extracts. Isoflavones such as genistin and genistein (and respective isomers) have been reported as having anti-inflammatory, antiproliferative, and antibacterial effects [36]. Also, phenolics like rutin, isoquercetin, and quercetin can play an important role as antimicrobial agents due to their capacity of interference on bacterial mechanisms of nucleic acid synthesis, cytoplasmic membrane, energy metabolisms, and being particularly effective against gram-positive bacteria [37] such as* S. aureus* studied in the current work. The antimicrobial activity of* P. tridentatum* extracts and in a less extension* M. pulegium* might be due to one of the mechanisms of action mentioned above. The richness of flavonols and isoflavones on* P. tridentatum* can be responsible for the increment in antibacterial activity exhibited for this extract compared to the* M. pulegium*. In fact, the high presence of compounds such as taxifolin, genistin, and biochanin often reported as having antibacterial activity [38] can be responsible for depletion of bacteria resistance mechanisms leading to increment in their susceptibility to these compounds. Also, it was observed that taxifolin and respective isomers extracted from* Hypericum japonicum* Thunb. ex. Murray (Guttiferae) delayed the protein synthesis of* S. aureus* (including the MRSA strains), affecting the synthesis of nucleic acids and enzymatic systems needed for bacteria growth [39]. This action is responsible for increasing the membranes permeability to drugs, leading to a decrease in bacteria survival, suggesting that these compounds might have a bacteriostatic effect rather than a bactericidal activity. Moreover it was noted that, in general, flavonoids and oligomers of flavonoids, particularly those with high grade of hydroxylation (such as flavonols, flavones, and flavanones), have a strong ability to link with bacteria cell walls from complexes [40, 41], affecting the bacteria growth and survival. Thus, plant extracts with high content of such compounds, like* P. tridentatum*, can be very useful when used in a complementary therapy with commercial drugs due to their bacteriostatic effect.

The majority of the studies available in literature about the antimicrobial activity of* P. tridentatum* and* M. pulegium* report mainly the effects of their essential oils (EOs) [19, 39–41] and very few about the effect of their hydroalcoholic extracts [41–43], and thus they attribute their antibacterial efficacy essentially to the EOs, and fewer conclusions are made about the importance of other bioactive compounds such as polyphenols. Moreover, their effects against* S. aureus* MSSA and MRSA have been scarcely explored. Thus, our results seem to be important because they not only reinforce the idea that richness of polyphenols is also critical for the antimicrobial capacity of any plant, but also show the bioactivities of these two herbs, proving that they can be used to extract bioactive compounds with antimicrobial activity against MSSA and MRSA.

## 4. Conclusion

*P. tridentatum* and* M. pulegium* are two important herbs from Mediterranean native flora and might be used to extract important and effective bioactive compounds against epidemiological important pathogenic bacteria, particularly against* S. aureus*, one of the most important pathogenic bacteria, often associated with foodborne outbreak diseases and hospital/clinical environment infections. Our results have shown that both extracts can be effective against MRSA and MSSA due to high content of different class of flavonoids, particularly flavonols, flavones, and isoflavones compounds, which can act synergistically with each other against those bacteria. Further research is needed to elucidate accurately the pathways and mechanisms used by these compounds against bacteria.

## Figures and Tables

**Table 1:** Isolates of *Staphylococcus aureus* used in the in vitro antibacterial assay.

Reference	Type	Source
MJMC021	Methicillin-resistant strains	Clinical
MJMC024	Methicillin-resistant strains	Clinical
MJMC026	Methicillin-resistant strains	Clinical
MJMC025	Methicillin-sensitive strains	Clinical
MJMC027	Methicillin-sensitive strains	Clinical
MJMC029	Methicillin-sensitive strains	Clinical

**Table 2:** Average content of polyphenols and respective retention time (Rt) and wavelengths of maximum absorption in the visible region (*λ*_max_) in *P. tridentatum *extracts (by elution order)^†^.

Polyphenols	Rt (min)	UV (nm)	UV-DAD/VIS bands (nm) in 70% methanol	Class	Mg·g^−1^ dry weight
Taxifolin	17.48	280	290, 327sh	Flavanol	21.76 ± 0.030
Genistin	19.79	320	259, 323sh	Isoflavone	16.75 ± 0.040
Genistein	22.73	320	259, 332sh	Isoflavone	12.01 ± 0.030
Quercetin-3-O-rutinoside	23.88	370	256, 266sh, 351	Flavonol	1.58 ± 0.030
Quercetin-3-O-glucoside	23.99	370	255, 266sh, 348	Flavonol	1.23 ± 0.010
Ferulic acid	24.32	320	244, 296sh, 324	Hydroxycinnamic acid	0.27 ± 0.002
Biochanin A-glucoside	30.66	320	265, 322sh	Flavone	1.37 ± 0.002
Apigenin	32.65	370	229, 267sh, 341	Flavone	0.44 ± 0.002
Biochanin A	34.88	320	265, 323sh	Flavone	2.89 ± 0.004

Total of hydroxycinnamic acids					0.27 ± 0.002
Total of flavonols					24.57 ± 0.100
Total of flavone					4.70 ± 0.008
Total of isoflavone					28.76 ± 0.070
Total of polyphenols identified					58.30 ± 0.180

^†^Values expressed as mean ± standard deviation of three replicates.

**Table 3:** Average content of polyphenols and respective retention time (Rt) and wavelengths of maximum absorption in the visible region (*λ*_max_) in *M. pulegium *extracts (by elution order)^†^.

Polyphenols	Rt (min)	UV (nm)	UV-DAD/VIS bands (nm) in 70% methanol	Class	Mg·g^−1^ dry weight
*p*-Hydroxybenzoic acid	13.46	280	227, 260sh, 295	Hydroxybenzoic acid	0.212 ± 0.004
Chlorogenic acid	17.95	320	242, 300sh, 327	Hydroxycinnamic acid	0.387 ± 0.004
(+)-Catechin	18.10	280	231, 280	Flavan-3-ols	0.212 ± 0.004
Caffeic acid	21.32	320	243, 296sh, 324	Hydroxycinnamic acid	0.230 ± 0.001
Quercetin-3-*O*-rutinoside	22.96	370	256, 266sh, 351	Flavonol	0.144 ± 0.003
Quercetin-3-*O*-glucoside	23.63	370	255, 266sh, 348	Flavonol	0.140 ± 0.002
Neohesperidin	24.84	280	283, 326	Flavanone	0.628 ± 0.001
Luteolin-7-*O*-glucoside	25.31	370	254, 266, 349	Flavone	0.201 ± 0.004
Diosmin	26.04	370	253, 268, 339	Flavone	0.623 ± 0.006
Rosmarinic acid	26.78	320	249, 299sh, 330	Hydroxycinnamic acid	0.287 ± 0.006
Ferulic acid	27.55	320	241, 296sh, 324	Hydroxycinnamic acid	0.277 ± 0.003
Apigenin	32.65	370	235, 267sh, 341	Flavone	0.323 ± 0.002

Total of hydroxybenzoic acids					0.212 ± 0.004
Total of hydroxycinnamic acids					1.181 ± 0.014
Total of flavan-3-ols					0.212 ± 0.004
Total of flavonols					0.284 ± 0.005
Total of flavones					1.147 ± 0.012
Total of flavanones					0.628 ± 0.001
Total polyphenols identified					3.664 ± 0.040

^†^Values expressed as mean ± standard deviation of three replicates.

**Table 4:** Minimum inhibitory concentration (MIC) of *P. tridentatum* and *M. pulegium *aqueous and methanolic extracts expressed as µg · mL^−1^^†^.

Isolate	Reference	Type	Gentamicin (commercial antibiotic)	*P. tridentatum*	*M. pulegium*
*S. aureus*	ATCC 13565	Standard	<39 (+++)	312.5 (++)	2500 (−)
*S. aureus*	MJMC021	MRSA	<39 (+++)	78.1 (+++)	2500 (−)
*S. aureus*	MJMC024	MRSA	<39 (+++)	78.1 (+++)	2500 (−)
*S. aureus*	MJMC026	MRSA	<39 (+++)	78.1 (+++)	2500 (−)
*S. aureus*	MJMC025	MSSA	<39 (+++)	39.1 (+++)	39.1 (+++)
*S. aureus*	MJMC027	MSSA	<39 (+++)	39.1 (+++)	78.1 (+++)
*S. aureus*	MJMC029	MSSA	<39 (+++)	39.1 (+++)	39.1 (+++)

^†^Inside brackets there is the classification of the antibacterial activity effect: strong (+++), if MIC values ≤ 100 *μ*g·mL^−1^, moderate (++) when 100 < MIC ≤ 500 *μ*g·mL^−1^, weak (+) when 500 < MIC ≤ 1000 *μ*g·mL^−1^, and null (−) (without effect) when MIC > 1000 *μ*g·mL^−1^.
